# DNA-Histone Cross-Links: Formation and Repair

**DOI:** 10.3389/fcell.2020.607045

**Published:** 2020-12-21

**Authors:** Manideep C. Pachva, Alexei F. Kisselev, Bakhyt T. Matkarimov, Murat Saparbaev, Regina Groisman

**Affiliations:** ^1^Department of Molecular Oncology, British Columbia Cancer Research Centre, Vancouver, BC, Canada; ^2^Department Drug Discovery and Development, Harrison School of Pharmacy, Auburn University, Auburn, AL, United States; ^3^National Laboratory Astana, Nazarbayev University, Nur-Sultan, Kazakhstan; ^4^Groupe “Mechanisms of DNA Repair and Carcinogenesis”, Equipe Labellisée LIGUE 2016, CNRS UMR 9019, Université Paris-Saclay, Villejuif, France

**Keywords:** DNA-histone cross-link, nucleosome core particle, chromatin, genome instability, spartan protease, proteasome, DNA repair

## Abstract

The nucleosome is a stretch of DNA wrapped around a histone octamer. Electrostatic interactions and hydrogen bonds between histones and DNA are vital for the stable organization of nucleosome core particles, and for the folding of chromatin into more compact structures, which regulate gene expression via controlled access to DNA. As a drawback of tight association, under genotoxic stress, DNA can accidentally cross-link to histone in a covalent manner, generating a highly toxic DNA-histone cross-link (DHC). DHC is a bulky lesion that can impede DNA transcription, replication, and repair, often with lethal consequences. The chemotherapeutic agent cisplatin, as well as ionizing and ultraviolet irradiations and endogenously occurring reactive aldehydes, generate DHCs by forming either stable or transient covalent bonds between DNA and side-chain amino groups of histone lysine residues. The mechanisms of DHC repair start to unravel, and certain common principles of DNA-protein cross-link (DPC) repair mechanisms that participate in the removal of cross-linked histones from DNA have been described. In general, DPC is removed via a two-step repair mechanism. First, cross-linked proteins are degraded by specific DPC proteases or by the proteasome, relieving steric hindrance. Second, the remaining DNA-peptide cross-links are eliminated in various DNA repair pathways. Delineating the molecular mechanisms of DHC repair would help target specific DNA repair proteins for therapeutic intervention to combat tumor resistance to chemotherapy and radiotherapy.

## Introduction

Cellular DNA is constantly altered by endogenous and exogenous factors, resulting in tens of thousands of lesions in a human cell every day (Lindahl, 1993). This damage may be classified into two types according to size: non-bulky DNA and bulky DNA. Non-bulky DNA lesions include base mismatches, abasic sites, and small base modifications, which in general are repaired by mismatch repair (MMR), base excision repair (BER), nucleotide incision repair (NIR), direct reversal repair (DRR), and translesion DNA synthesis (TLS) (Gros et al., 2004; Fortini and Dogliotti, 2007; Sharma et al., 2013; Yi and He, 2013; Ignatov et al., 2017). Bulky DNA lesions include, among other types of damage: double-strand breaks, DNA-protein cross-links (DPCs), and intra- and inter-strand DNA cross-links. The structural complexity of certain bulky DNA lesions requires the use of several DNA repair pathways acting in a coordinated manner, including homologous recombination (HR), non-homologous DNA end-joining (NHEJ), nucleotide excision repair (NER), TLS and BER; Fanconi anemia (FA) signaling system and complex proteolytic machinery (Ishchenko et al., 2006; Ho and Schärer, 2010; Duxin et al., 2014; Tretyakova et al., 2015; Martin et al., 2017). Non-bulky DNA lesions cause limited and local DNA perturbations, whereas bulky ones induce significant distortions in the overall DNA helix structure (Ide et al., 2011). DNA-protein cross-links (DPCs) are formed when a protein covalently binds to DNA (Tretyakova et al., 2015). They are difficult to repair because of their super-bulky character compared with known voluminous, helix-distorting DNA lesions, such as UV-induced pyrimidine dimers. These super-bulky adducts can be generated by exposure of cells to endogenous and exogenous cross-linking agents (Stingele et al., 2017; Zhang et al., 2020). The presence of protein covalently attached to DNA strongly interferes with DNA replication, transcription, repair, and chromatin remodeling (Kuo et al., 2007; Klages-Mundt and Li, 2017; Yudkina et al., 2018; Ji et al., 2019). DPCs may be classified into five types, according to the nature of the covalent link in the DNA-protein complex and the presence of DNA strand breaks (Ide et al., 2015, 2018; Nakano et al., 2017). Type 1, the most common type of DPC, is formed when proteins covalently link to a nitrogenous base in undisrupted DNA. Type 2-4 cross-links occur when DNA-cleaving enzymes are trapped in a covalent intermediate with a DNA strand (Ide et al., 2015, 2018; Nakano et al., 2017). Type 2 is formed when bi-functional DNA glycosylases and repair enzymes containing β-lyase activity such as DNA polymerase β and Parp1 irreversibly bind to a cleaved apurinic/apyrimidinic (AP) site (Ide et al., 2015, 2018; Nakano et al., 2017). Type 3 is generated during abortive DNA strand cleavage by topoisomerase 1 (Top1) and formation of a covalent tyrosinyl–phosphodiester bond between the protein and the 3′-terminal DNA phosphate moiety of SSB (Ide et al., 2015, 2018; Nakano et al., 2017). The abortive action of topoisomerase 2 (Top2) generates type 4 DPC, in which tyrosine is linked to the 5′-terminal phosphates of double-strand breaks (DSB) (Ide et al., 2015, 2018; Nakano et al., 2017). Recently, a new type of DPC emerged after the discovery of HMCES, a 5-hydroxymethylcytosine (5hmC) binding protein which can recognize abasic sites in single stranded DNA (ssDNA) and form a covalent ssDNA-HMCES crosslink to prevent error-prone translesion synthesis past the lesion (Mohni et al., 2019). Because of the differences in structure and composition between these five groups, each type of DPC is processed by a distinct repair mechanism. It seems difficult to remove super-bulky Type 1 DPC in the canonical linear DNA excision repair pathways because the presence of a protein molecule blocks access to DNA. Nevertheless, recent studies have revealed that nucleotide excision repair (NER) and homologous recombination (HR) can remove certain types of DPCs in a nuclease-dependent manner (Zhang et al., 2020). However, it is still not clear whether these repair pathways could deal with other types of DPC. Stingele et al. (2017) have proposed that each constituent of DPC: DNA, protein, and the covalent linkage between them might be processed by three different repair mechanisms. A recent paper by Kühbacher and Duxin (2020) provides comprehensive review on the formation and repair of DPCs. In this review, we summarize the current knowledge regarding the repair mechanisms involved in removal of DHCs induced by various genotoxic agents. Covalent cross-linking to DNA occurs more often with DNA binding proteins, such as histones, transcription factors, and DNA metabolizing enzymes including repair factors and topoisomerases (Klages-Mundt and Li, 2017). In the cell nucleus, histones are assembled into an octamer forming the nucleosome core with 147 bp of DNA wrapped around and tightly bound to it (Luger et al., 1997, 2012). This basic chromatin structure makes histones primary targets of DNA cross-linking agents, leading to the formation of DNA-histone cross-links (DHC) (Solomon and Varshavsky, 1985). Currently, the repair mechanisms counteracting DHCs generated by various factors only started to unravel.

### DNA-Histone Cross-Links (DHCs)

Nucleosomal DNA is packaged into compact units referred as chromosomes, in which core nucleosome particles are connected by stretches of linker DNA up to 80 bp length. A nucleosome core particle (NCP) is composed of two copies each of histones H2A, H2B, H3, and H4. The molecular weight of individual histones range from 11 to 22 KDa, whereas the molecular weight of histone octamer in NCP is 210 KDa (Eickbush and Moudrianakis, 1978; Luger et al., 1997). The stability of the nucleosome is based on various protein-protein interactions, and numerous non-covalent electrostatic and hydrogen bonds between histones and the DNA duplex (Luger et al., 1997, 2012; Davey et al., 2002; Rohs et al., 2009). The primary structure of chromatin can be depicted as a beads-on-a-string organization of individual nucleosomes, which can be further folded into compact secondary and tertiary structures, with the help of histone variants present in certain nucleosomes and post-translational modifications (PTMs) situated in disordered histone tails (Woodcock and Dimitrov, 2001; Luger et al., 2012). The folding of chromatin into primary, secondary, and tertiary structures is crucial for regulating the accessibility of DNA to complex multi-protein machinery involved in DNA replication, transcription, and repair. Non-covalent interactions between DNA and histones enable chromatin dynamics to switch between the closed and open conformations. DHCs impair chromatin flexibility, which may subsequently affect long-distance interactions in chromatin that would indirectly disturb DNA replication, transcription, and repair within a topologically associating domain (TAD) (Hinz et al., 2010; Todd and Lippard, 2010; Tretyakova et al., 2015; Hauer and Gasser, 2017; Nakano et al., 2017). DHCs belong to type 1, a non-enzymatic form of DPC, in which a protein is covalently attached to an undisrupted DNA (Ide et al., 2011). Several comprehensive studies describing the mechanisms of formation of DHCs have been published recently (Ming et al., 2017; Shang et al., 2019; Yang and Greenberg, 2019), nevertheless, it is not known whether specific repair mechanisms for the removal of DHCs exist. In this review, we focus mainly on the repair pathways of DHCs and briefly describe their formation.

### Formation of DHCs

**FIGURE 1:**
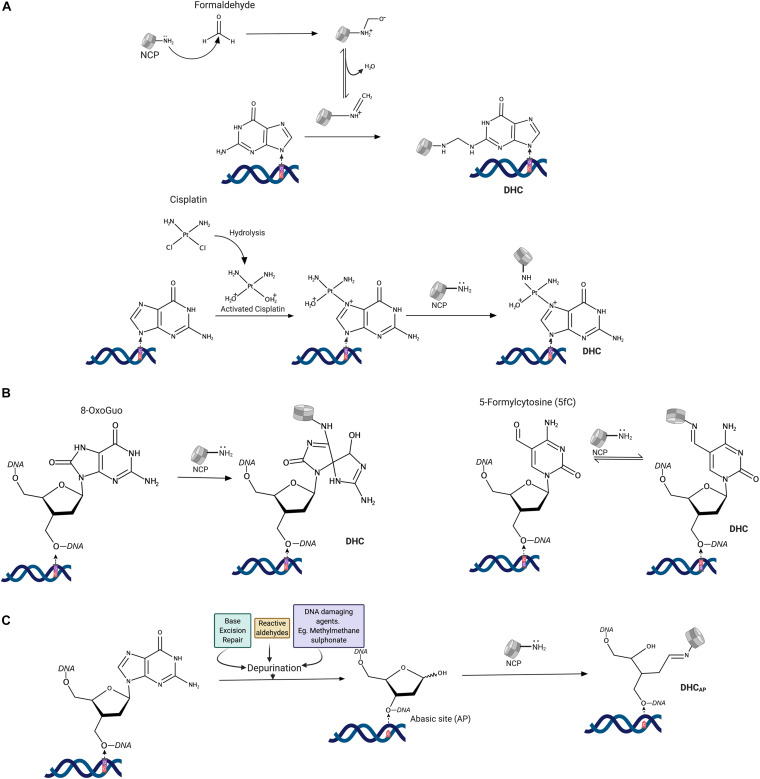
Mechanisms of histone-DNA cross-links formation. **(A)** Reaction mechanisms of DNA cross-linking agents. **(B)** Direct cross-linking of histones to modified DNA bases. **(C)** Abasic site mediated cross-linking of histones to DNA. NCP-NH_2_: *N*-terminal amine (Lysine) of histones in the nucleosome core.

A water-soluble covalent complex of DNA and histones (H2A and H2B) was first identified in a UV cross-linking assay (Smith, 1966; Sperling and Sperling, 1978). With this finding, it became evident that UV irradiation can induce DHCs in addition to well-known pyrimidine dimers. It was then discovered that exogenous and endogenous aldehydes could also form DHCs in cells (Lam et al., 1985; Kuykendall and Bogdanffy, 1992). More than 10% of amino acid residues in histones are lysines, whereas, aldehydes preferentially react with ε-amino groups of lysine side-chains with the formation of a Schiff base, which further reacts with exocyclic amino groups of guanine, adenine, and cytosine DNA bases, creating methylene linkage. Many cross-linking agents, such as chromate, metal ions, and cisplatin (*cis*-diaminedichloroplatinum-II), also induce DHCs in cells (Zhitkovich and Costa, 1992). Platinum compounds not only cause DNA-DNA cross-links but also covalently link DNA-protein complexes. In the case of histones (Figure 1A), these compounds cross-link ε-amino-groups of lysines and N^7^ atoms of guanosines (Tretyakova et al., 2015; Ming et al., 2017). Cross-links between DNA and methionine residues were also observed in an X-ray structure of nucleosomes treated with platinum compounds (Wu et al., 2008). Exposure of purified nucleosome to bi-functional alkylating agents (e.g., nitrogen mustards) also cross-links histones to guanosines in DNA (Shang et al., 2019); however, these types of cross-links in cells are much less abundant than DNA cross-links with cysteines and histidines of non-histone proteins (Loeber et al., 2009).

Histones can also directly react with 5-formylcytosine, a naturally occurring modified DNA base, and 8-oxoguanine, a major oxidative DNA damage product. Lysine amino groups react with 5-formylcytosine (Figure 1B), with the formation of a reversible Schiff base (Li et al., 2017; Raiber et al., 2018). The reaction of lysine side-chains with 8-oxoguanosine produced a stable protein cross-linked spiroiminodihydantoin (Sp) adduct (Xu et al., 2008).

Finally, the majority of DHCs are produced by a reaction between histone lysines and an aldehyde form of the 2′-deoxyribose at apurinic/apyrimidinic (AP) sites (Figure 1C) that are either directly formed upon damage or generated during excision of damaged bases in the base excision repair pathway (Solomon and Varshavsky, 1985; Sczepanski et al., 2010). The resulting Schiff base often undergoes strand-breaking ß-elimination, followed by a reversal of a histone-DNA cross-link. Since histone emerges unaltered from the reaction, the whole process is sometimes referred to as histone-catalyzed strand cleavage at AP sites (Ren et al., 2019). It should be noted that histone PTMs and the chromatin state could have a significant impact on DHC formation at abasic sites and with DNA bases (Sczepanski et al., 2010; Bowman and Poirier, 2015).

### Mechanisms of Repair of DHC

Although DPCs, especially DHCs, often occur in cells and present a constant threat to genome stability, it is presumed that, except for tyrosyl-DNA phosphodiesterases, there is no specialized DNA repair pathway dedicated to meet these super-bulky challenges. Instead, the cell employs several distinct DNA repair and protein degradation mechanisms to target cross-linked DNA and protein/histone components in a given DPC/DHC. The covalently bound protein could be detected and degraded to a small peptide by cell proteolytic machinery, such as the specialized proteases SPRTN/Wss1, Ddi1, and GCNA1, or by proteasome, an ATP-dependent multi-subunit protease complex, whereas the damaged DNA component is detected and repaired in the NER, BER, HR, NHEJ, and FA pathways.

### Proteasome-Dependent Proteolysis of Histones Cross-Linked to DNA

Proteasome-mediated proteolysis is the major pathway for the degradation of damaged proteins in a cell. A 26S proteasome consists of a cylindrical 20S core particle and one or two 19S regulatory particles (Ciechanover, 1998; Lecker et al., 2006). Although 20S core can bind to different regulatory particles, only the 19S particle confers the ability to degrade ubiquitylated proteins (Coux et al., 1996; Adams, 2004; Stadtmueller and Hill, 2011). Considering the vital role of the proteasome in the degradation of damaged protein, proteasome and ubiquitin involvement in the proteolysis of DHCs or DPCs remains a topic of debate. Inhibition of proteasome in *Xenopus* egg extracts did not stabilize the DPCs (Nakano et al., 2007; Duxin et al., 2014). However, many studies of the repair of DPCs in mammalian cells suggest proteasome participation (Adams, 2004; Baker et al., 2007; Zecevic et al., 2010; Larsen et al., 2019). Proteasome involvement in DHC removal surfaced for the first time in the research of Quievryn and Zhitkovich (2000), who discovered that proteasome inhibitors prevent the removal of DHCs and sensitize human cells to lower levels of formaldehyde. A study in *Xenopus* egg extracts found that DPCs are ubiquitylated by TRAIP E3 ubiquitin ligase and are subsequently degraded by the proteasome (Duxin et al., 2014; Larsen et al., 2019). However, an earlier study clearly demonstrated that DPCs are not marked with polyubiquitin chains, but are nevertheless subjected to proteasomal degradation by a mechanism that is not well understood (Nakano et al., 2009). The 26S proteasome can degrade purified non-ubiquitylated histones (Kisselev et al., 2006), raising the possibility of proteasomal degradation of non-ubiquitylated damaged histones in cells. A couple of studies have demonstrated that during replication stress induced by genotoxic agents, histones are hyperacetylated, and then specifically degraded in a ubiquitin-independent manner by a complex of 20S proteasome with PA200 proteasome activator, a distinct regulatory particle (Qian et al., 2013; Mandemaker et al., 2018). Although these studies have demonstrated that the ubiquitin-independent degradation of acetylated histones alleviates replication stress, the additional function of PA200-20S proteasome in DHC repair cannot be excluded. Moreover, PA200 was detected in nuclear speckles, and its role in DNA repair has been proposed (Ustrell et al., 2002). Thus, more detailed understanding of the role of proteasome in DHC repair requires further investigation.

The 20S proteasome is a hollow, barrel-shaped particle composed of 28 non-identical subunits arranged into four stacked rings. The active sites are sequestered inside an internal cavity separated from regulatory 19S and PA200 complexes by a gated channel. This 13Å channel is too narrow for a folded protein to enter (Löwe et al., 1995; Groll et al., 1997). For complete degradation of a DNA-cross-linked protein, the cross-linked DNA nucleotide itself would have to enter the proteolytic chamber, pulling a DNA strand inside. However, the DNA component of a DPC might be too bulky to enter the channel. Therefore, proteasome can remove only part of a cross-linked protein, converting DHC into a smaller DNA-peptide cross-link. Alternatively, traditional proteases, in which an active site is located in a cleft on the enzyme surface, could be involved in excision of the bulk of the non-cross-linked polypeptide chain, which can then be degraded by any of these proteases and by the proteasome.

## Metalloprotease-Based Proteolysis of Histones Cross-Linked to DNA

**FIGURE 2:**
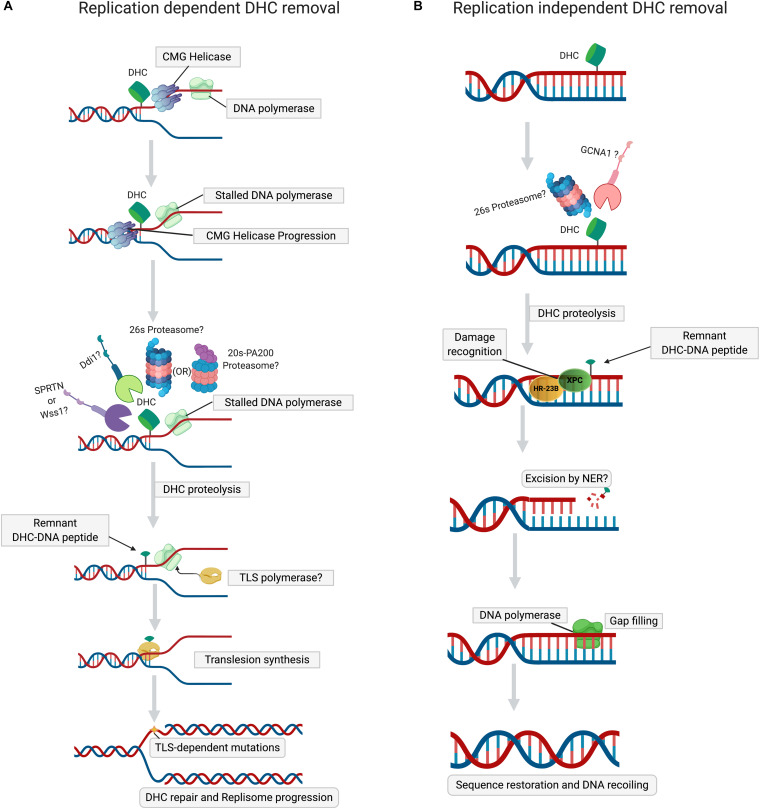
Mechanisms of DHC repair. **(A)** Replication-dependent DHC removal. Stalling of DNA polymerase at DHC during replication triggers proteolysis of DHC by the proteasome (26S or PA200), followed either by the SPRTN or Ddi1 proteases. The peptide-DNA crosslink, a product of proteolyzed DHC, is bypassed by translesion synthesis (TLS) DNA polymerases, resuming the replisome progression. **(B)** Replication-independent DHC removal. In non-dividing cells, when histones are cross-linked to DNA, distortion of the DNA helix induces histone PTMs (acetylation or SUMOylation) and chromatin opening, thus permitting the proteolysis of DHC by 26S proteasome or GCNA1 protease. The peptide-DNA crosslink, a remnant of proteolyzed DHC, is then excised by NER, followed by gap-filling by DNA polymerase and ligation, which lead to the restoration of primary DNA sequence and chromatin.

Wss1 (weak suppressor of smt3) is a DNA-dependent metalloprotease, whose role in DPC removal was unraveled in a study in which treatment of yeast cells lacking Wss1 and TDP1 with formaldehyde resulted in a synthetic sickness (Stingele et al., 2014). It was also demonstrated that SUMOylation of both enzymatic and non-enzymatic DPCs by DNA-bound SUMO ligases targets them to Wss1 (Psakhye and Jentsch, 2012; Jentsch and Psakhye, 2013; Stingele et al., 2014; Balakirev et al., 2015). Search for an ortholog of Wss1 in higher eukaryotes revealed another metalloprotease, Dvc1/Spartan, of the SprT protease family, whose function was initially thought to be a removal of Polη from chromatin during translesion DNA repair synthesis. (Davis et al., 2012; Mosbech et al., 2012). Spartan/Dvc1 has a similar domain organization and shares a common evolutionary origin with Wss1. Like Wss1, Dvc1/Spartan also repairs DPCs in *Xenopus* egg extracts (Duxin et al., 2014; Stingele et al., 2014, 2015). A detailed study of DPC removal by Spartan/Dvc1 in *Xenopus* levis revealed stepwise proteolysis of DPC during DNA replication (Larsen et al., 2019). During replication, on both the leading and lagging strands, CMG helicase bypasses the DPC lesion, followed by the stalling of DNA polymerase (Duxin et al., 2014; Larsen et al., 2019; Sparks et al., 2019). Replicative DNA polymerase stalled at DPC triggers the recruitment of Spartan protease (Figure 2A), which then degrades the DPC protein component (Larsen et al., 2019; Reinking et al., 2020). However, in the case of DHCs, CMG helicase bypass would depend on the complexity of nucleosomal damage. If the crosslink involves just a single histone within NCP, then CMG helicase can bypass the DHC due to small size of protein residue. If multiple histones within NCP are cross-linked, then the large nucleosome-size DHC should stall CMG helicase, triggering the ubiquitination of crosslinked histones by TRAIP E3 ubiquitin ligase and their subsequent degradation by 26S proteasome (Nakano et al., 2013; Larsen et al., 2019; Sparks et al., 2019). In contrast to Wss1, targeting of DPCs by Spartan/Dvc1 does not require SUMOylation of DPC (Duxin et al., 2014; Larsen et al., 2019). Several studies have demonstrated that proteasome and Spartan might work in concert to attain efficient DPC proteolysis. For proteolysis of DPC by proteasome, the active site topology in the 20S core particle makes it arduous for efficient proteolysis, leaving a larger peptide adduct, which could be further degraded by Spartan (Larsen et al., 2019; Sparks et al., 2019). The above observations support a hypothesis proposed by the Walter laboratory (Larsen et al., 2019) that initial proteolysis of DPC by proteasome may help reduce the size of a DPC for the CMG helicase bypass. This stepwise process applies for most type 1 DPCs when histones cross-linked to DNA are initially degraded either by 26S proteasome or PA200-20S proteasome, and in a second step by Spartan (Figure 2A). Validation of this hypothetical order of steps in proteolysis of DHC by the proteasome and Spartan requires more research. However, a recent study proved that Wss1 is actively involved in histone proteolysis in a NCP during replication stress (Maddi et al., 2020). Although this study showed that Wss1 removes histones non-covalently bound to DNA, it is very likely that this protease can also remove histones covalently cross-linked to DNA. Initially, it was thought that Spartan/Dvc1 could only execute DPC proteolysis in a replication-dependent manner, but later it was demonstrated that Spartan/Dvc1 could act on a single-stranded DNA to degrade DPC independently of replisome (Larsen et al., 2019). A couple of other studies have also demonstrated that in fly embryos Spartan is recruited to chromatin before replication and that its absence greatly sensitized the arrested, non-replicating L1 worm larvae to formaldehyde (Delabaere et al., 2014; Stingele et al., 2016; Reinking et al., 2020). This evidence clearly defines a role for Spartan in the replication-independent repair of DPCs, which may be required for chromatin-based transactions associated with transcription. More data are required to confirm the existence of transcription-coupled DPC repair in cells.

Germ cell nuclear acidic peptidase (GCNA), also known as germ cell nuclear antigen or acidic repeat containing (ACRC), is a metalloprotease that contains an SprT domain, such as Spartan/Dvc1 (Carmell et al., 2016; Dokshin et al., 2020). It has recently been demonstrated in *Caenorhabditis elegans* that GCNA/ACRC, with its C-terminal Spartan (SprT)-domain, can also be involved in the removal of formaldehyde-induced DPCs in exposed cells (Borgermann et al., 2019). Recently, another study of Drosophila embryos clearly established that the loss of GCNA resulted in the accumulation of DPCs that included histones, confirming the role of GCNA in the removal of DHCs (Bhargava et al., 2020). Like Wss1, it was proved that SUMOylation of DPC triggers the recruitment of GCNA/ACRC protease, but in a replication-independent manner (Borgermann et al., 2019). Noteworthy, the chromatin-associated proteins were highly SUMOylated (Borgermann et al., 2019) (Figure 2B), suggesting that formaldehyde induces DHCs, which are then SUMOylated to recruit GCNA/ACRC SprT protease. However, the detailed mechanism of GCNA/ACRC-mediated repair of DHC requires further investigation.

## Ddi1 Protease-Based Proteolysis of Histones Cross-Linked to DNA

It was recently shown that a novel protease, Ddi1 (DNA-damage inducible-1), is involved in DPC repair (Serbyn et al., 2020). Ddi1 is an aspartic protease. Its protease domain is structurally similar to that of retroviral aspartic proteases (Krylov and Koonin, 2001; Sirkis et al., 2006). Ddi1 is involved in response to hydroxyurea (HU)-induced replication stress by facilitating the removal of replication termination factor RTF2, restarting stalled replication forks (Kottemann et al., 2018). In addition, it is well documented that *Saccharomyces cerevisiae* Ddi1 contains a ubiquitin-like (UBL)-ubiquitin associated (UBA) domain, which enables the protein to act as a shuttle delivering ubiquitylated proteins to the proteasome (Dantuma et al., 2009). However, a genetic screen using the *tdp1wss1* mutant defective in the processing of DNA-Top1 covalent cross-link (Top1cc), revealed that Ddi1 could rescue mutant cells treated with Top1cc trapping and other genotoxic agents (Serbyn et al., 2020). Retention of Flp-Nick-Induced DPC in Ddi1-depleted cells confirmed a direct role of Ddi1 in DPC repair, due to proteolysis catalyzed by its retroviral-like protease domain (Krylov and Koonin, 2001; Serbyn et al., 2020). Previous evidence supports the idea that the proteolytic function of Ddi1 in DNA repair is replisome-dependent (Clarke et al., 2001; Kottemann et al., 2018) (Figure 2A). However, Ddi1-dependent proteolysis of stalled RNA polymerase II (Pol II) on chromatin suggests an additional function of this protease (Serbyn et al., 2020). Interestingly, it has been demonstrated that Ddi1-mediated proteolysis of DPCs can be a proteasome-independent compensatory mechanism in the case of Wss1 dysfunction (Serbyn et al., 2020). Yeast Ddi1, and the human ortholog DDI2, are more dependent on poly-ubiquitination as compared to proteasome; these proteases cannot cleave non-ubiquitylated proteins and prefer substrates bearing extra-long ubiquitin chains (Sivá et al., 2016; Dirac-Svejstrup et al., 2020; Yip et al., 2020). Serbyn et al. (2020) found that depletion of Ddi1 makes cells hypersensitive to formaldehyde, suggesting that DHC can be a major substrate of aspartyl protease-catalyzed proteolysis. However, the extension of this function of yeast Ddi1 to its human DDI2 ortholog remains unclear, since certain structural domains of Ddi1 and human DDI2 are not evolutionarily conserved (Sivá et al., 2016; Trempe et al., 2016). In conclusion, the respective roles of Ddi1 and DDI2 in the removal of DHCs in higher eukaryotes remain to be investigated.

## DNA Repair Pathways in DHC Removal

As mentioned earlier, protein components (histones) of DHC are mainly targeted by specific proteases; however, partial proteolysis of cross-linked histones will still leave a small peptide covalently attached to DNA, requiring the coupling of proteases with classical DNA repair pathways to ensure complete removal of DHC and restoration of the primary DNA structure. In absence of studies on the DHC specific repair mechanisms, in this review we tried to infer the knowledge available on specific type 1 DPC repair mechanisms that are applicable to DHCs.

### Nucleotide Excision Repair

Several biochemical and genetic studies showed that NER-deficient cells are sensitive to formaldehyde, suggesting direct involvement of NER in DHC removal (Nishioka, 1973; Takahashi et al., 1985). However, other studies have revealed that NER defective cells are not sensitive to 5-aza-2′-deoxycytidine (5-azadC)-based treatment, which induces cross-linking of DNA cytosine residues to DNA methyltransferase 1 (Dnmt1). This implies that NER can excise DPCs with smaller proteins (<8–12 kDa) induced by formaldehyde, but does not excise DPCs that contain proteins of larger size (>15 kDa), induced by 5-azaC (Bhagwat and Roberts, 1987; Lal et al., 1988; Nakano et al., 2007). This size limitation for excision of DPCs by bacterial NER may be attributed to the strong dependence of UvrB loading efficiency on DPC size, which subsequently influences the overall incision efficiency of the UvrABC nuclease complex. Thus, bacterial NER machinery can make bracketed incisions on DNA strands containing DPC with a small peptide, but not with a large one (Minko et al., 2002, 2005; Nakano et al., 2007). Histones are relatively small proteins ranging in size from 11 to 16 kDa; it was therefore proposed that DHCs could be removed in the NER pathway without preceding proteolysis step. Based on these observations, one may also propose that DHCs/DPCs induced by AP sites in DNA might be removed by NER (Torres-Ramos et al., 2000; Sczepanski et al., 2010). However, the role of NER in repair of AP sites induced DHCs remains to be established. A few studies have demonstrated that NER is implicated in DPC repair in mammalian cells (Fornace, 1982; Baker et al., 2007). Each histone is very small in size; they are assembled into a large NCP, which may sterically inhibit the loading of XPC/Rad23B and XPA/RPA complexes onto the DHC site (Ide et al., 2011). When the XPA/RPA complex fails to bind to the damage site, TFIIH cannot access and open up the DNA duplex around DHC to discriminate between intact and damaged DNA strands. All these render subsequent cleavage of this strand by XPG and XPF-ERCC1 nucleases unfeasible. Thus, proteolysis of DNA-cross-linked NCP might be required for efficient removal of a histone trapped on DNA by the NER machinery (Nakano et al., 2009). A study using mammalian cells has demonstrated that XPF/ERCC1 nuclease requires pre-processing of the cross-linked protein adduct by the proteasome and proteases before its removal (Nakano et al., 2009; Zhang et al., 2011; Stingele et al., 2017). In *uvrA* cells, plasmid cross-linked to the partially digested histone H1 (peptide sizes: 4.5 kDa and 1.8–3.5 kDa) was more efficiently repaired than a DPC containing a full-length histone (22 kDa) (Nakano et al., 2007) (Figure 2A). It is also worth noting that chromatin remodeling at a DNA damage site is required to provide access to NER machinery (Dinant et al., 2012). Covalent cross-linking of histones will impede this process, strongly suggesting that shrinkage of the bulky protein component must precede repair of the DNA component by the NER pathway. Further studies are required to understand the involvement of specific repair pathway(s) that precede and succeed NER and the sequence of steps involved in the removal of DPC/DHC (involving NER).

### Homologous Recombination

The role of homologous recombination in DPC repair has been addressed in bacterial genetic studies, where it was found that *Escherichia coli recA* and *recB* mutants defective for homologous recombination (HR) are sensitized to formaldehyde and 5-azadC-induced DPCs (Nishioka, 1973; Takahashi et al., 1985). Further studies revealed that the role of HR in DPC repair is highly conserved in mammalian cells, and that clipping of DPC by the conventional MRN complex leads to strong resistance to DPC-inducing agents (Connelly et al., 2003; Neale et al., 2005; Ridpath et al., 2007; de Graaf et al., 2009; Orta et al., 2013). MRN, a heterotrimeric protein complex, is a DNA nuclease involved in the resection of a double-strand break (DSB) that initiates the HR pathway (Neale et al., 2005; Rothenberg et al., 2009). Increased sister chromatid exchange rates and accumulation of DSBs and RAD51 foci near DPC in formaldehyde-treated mammalian cells further support involvement of HR pathway in DPC removal (Shaham et al., 1997). However, the molecular mechanism of HR-mediated DPC removal is still poorly understood. The repair of DPC in *E. coli* is dependent on the RecBCD nuclease that initiates the HR pathway (Bidnenko et al., 2002; Nakano et al., 2007). DSB adjacent to DPC and stalled replisome could be generated by re-replication of incomplete nascent DNA strands, with the subsequent collapse of the replication fork. They can also be generated by RecG helicase-mediated fork reversal, leading to the formation of a Holliday junction, which is then processed by RecBCD nuclease to generate DSB (McGlynn and Lloyd, 2001; Michel et al., 2004; Payne et al., 2006; Nakano et al., 2007, 2009). Certain studies suggest that replisome stalling at DPC does not lead to fork collapse, because template switching via fork reversal may allow DNA synthesis (Nakano et al., 2007; Stingele et al., 2015; Klages-Mundt and Li, 2017). In addition, other studies showed that DSB formation is not observed during the replication-dependent repair of DPCs in *Xenopus* egg extracts and hamster cells treated with DPC-inducing agents (Speit et al., 2000; Duxin et al., 2014). Thus, studies regarding the induction of DSBs near DPC have provided conflicting results, necessitating further studies of the mechanisms of HR-mediated DPCs repair. Differences in the sensitivity of *E. coli recA* and *uvrA* mutants, deficient for HR and NER respectively, to large DPCs inducing 5-azadC, revealed that unlike NER, HR could repair large DPCs (Santi et al., 1984; Bhagwat and Roberts, 1987; Ide et al., 2011; Zhang et al., 2020). Since histones are small proteins (11–16 kDa), their size should not be an obstacle to DHC repair in the HR-pathway. However, HR may not directly repair the DHC lesion in the chromatin context until access is provided to DNA damage-sensing factors. Thus, pre-processing of NCP containing a DHC or a repair pathway that precedes HR must be required to repair DHC by HR. Further research is needed to validate this model.

### Fanconi Anemia Pathway

The Fanconi anemia (FA) pathway is involved in the repair of inter-strand DNA cross-links (ICLs) and plays a pivotal role in cellular defense against reactive aldehydes (Ridpath et al., 2007; Rosado et al., 2011; Kottemann and Smogorzewska, 2013; Ceccaldi et al., 2016). However, the role of the FA pathway in DPCs repair is currently under debate (Duxin and Walter, 2015). A study conducted by Orta et al. (2013) suggests that Fanconi anemia-dependent HR is required for DPC removal in cells treated with 5-azadC. In addition, several studies have shown that cells depleted in FANC2, FANCQ/XPF, and FANCG proteins are sensitive to DPC-inducing agents, such as acetaldehyde, formaldehyde, and 5-azadC (Ridpath et al., 2007; Mechilli et al., 2008; Lorenti Garcia et al., 2009; Langevin et al., 2011; Rosado et al., 2011; Orta et al., 2013). The majority of DPCs induced by reactive aldehydes are DHCs (Solomon and Varshavsky, 1985). These results are in stark contrast to an *in vitro* study by Duxin et al. (2014), who demonstrated that depletion of FANCI-FANCD2 from *Xenopus* egg extracts inhibited ICL repair, but not DPC repair or TLS-mediated bypass. In agreement with latter observations, studies using *C. elegans* and mouse embryonic fibroblasts showed that depletion of FANCD2 did not affect the cells’ sensitivity to formaldehyde (Stingele et al., 2016). Despite these conflicting reports, the potential role of FA pathway components in the removal of DHCs should not be overlooked. A conserved FANCM-MHF DNA remodeling complex that recognizes a DNA lesion at the stalled replication fork, contains histone-binding sites (Yan et al., 2010). Therefore, it is possible that the FANCM-MHF complex, which recruits downstream Fanconi proteins to excise DHC, could readily recognize DNA cross-linked histones. Future studies are necessary to confirm and delineate the role of the FA pathway in DHC removal, and to determine whether the FA pathway requires pre-processing of DHC/DPC for their removal.

### Base Excision Repair

DNA glycosylase-initiated base excision repair (BER) is a major pathway for removing small non-bulky base lesions resulting from deamination, oxidation, and alkylation which do not significantly distort the DNA helix (Krokan and Bjørås, 2013). However, the DNA glycosylases NEIL1 and NEIL3 can also resolve psoralen-induced bulky ICLs in three- and four-stranded DNA structures (Couvé-Privat et al., 2007; Couvé et al., 2009; Martin et al., 2017). NEIL1, a bi-functional DNA glycosylase, can also repair Sp-amine adduct-containing DN-protein cross-links (McKibbin et al., 2013). Furthermore, another study demonstrated that a DPC generated as repair intermediate of PARP1, could be processed by BER machinery (Prasad et al., 2019). Thus, the role of BER may not be limited to small non-bulky DNA lesions.

Interestingly, it has been found that an oxidized AP site formed by a reactive oxygen species (ROS) can trap DNA polymerase β (Polβ) to form a stable DNA- Polβ cross-link (Polβ-DPC) (DeMott et al., 2002). It was later found that DNA glycosylase-generated AP sites can also trap several DNA repair proteins, such as PARP1, Ku proteins, DNA polymerase λ (Polλ), and other factors (Prasad et al., 2019; Quiñones et al., 2020). Thus, under certain circumstances, instead of repairing the lesion, BER can act as a source of a DPC lesion.

It is unknown whether impairment of BER machinery affects cells’ sensitivity to various DPC inducing agents. Also, it is not clear whether BER, like NER, has a size limit for processing of DPCs. As mentioned earlier, histones in a NCP can cross-link to abasic sites. In fact, the AP site generated by a DNA glycosylase may trap histones to form a covalent DHC. A Schiff base at an AP site can lead to DNA strand scission, thus contributing to major nucleosomal DNA damage. Although detailed information on the role of BER in formation of DHC is available, at present, little is known about the role of DNA glycosylases and AP endonucleases in the repair of this chromatin damage. Past evidence suggests that NEIL1 DNA glycosylase has a flexible active site that can accommodate bulky modifications of DNA bases and efficiently remove them (Couvé et al., 2009; McKibbin et al., 2013). Thus, it is predicted that NEIL1, and possibly NEIL3, may play a role in the processing of DHC, but that remains to be confirmed. Also, several studies suggest that BER coupled to proteolysis could participate in the efficient removal of DPC/DHC (Hauer et al., 2017; Prasad et al., 2019; Quiñones et al., 2020). Hence, further studies are required to gather information on the role of BER proteins in DHC removal.

### Translesion Synthesis

When a DNA replication fork stalls at unrepaired DNA damage, the cell can circumvent the obstacle through tolerance pathways, such as an HR-mediated template switch mechanism and translesion DNA synthesis (TLS). TLS is a lesion bypass mechanism that tolerates DNA damage and allows for DNA replication to proceed through unrepaired bulky nucleobase adducts (Woodgate, 1999; Friedberg et al., 2005; Lehmann et al., 2007; Sale, 2013). DPCs are super-bulky lesions that can stall replication forks and lead to their collapse. These lesions require a collaborative network of several cellular repair systems to remove them. However, removal of DPC in a stalled replication fork could provoke fork collapse and generation of DSB, with detrimental consequences to a cell, depending on the DNA repair mechanism used. On the other hand, the TLS pathway initiated by specific DNA polymerases replicates over and past the lesion in the damaged DNA template, thus providing a form of DNA damage tolerance, which avoids fork collapse and DSB. Therefore, TLS can play an important role in the management of DPCs in cells. Indeed, several studies have reported that specialized TLS DNA polymerases can bypass DPCs (Duxin et al., 2014; Wickramaratne et al., 2016; Pande et al., 2017; Larsen et al., 2019). Nevertheless, these studies demonstrated that large non-processed DPCs require the degradation of large protein adducts to smaller peptides cross-linked to DNA to allow bypass by TLS DNA polymerases. Thus, partial proteolysis of DPCs is necessary for efficient TLS bypass (Figure 2A). Larsen et al. (2019) study in *Xenopus* egg extracts clearly established that DPC proteolysis by Spartan/Dvc1 ensures efficient bypass by TLS-specific DNA polymerases REV1-Polζ. Although TLS DNA polymerases help bypass the DPC lesion, the extension of DNA past the lesion by the same DNA polymerases is error-prone and can lead to mutagenesis. Hence, DNA repair pathways, such as mismatch repair (MMR) and NER, could be coupled with TLS to prevent DPC-induced mutations. A study by Wickramaratne et al. (2016) demonstrated that partially digested DHC (histones H4 and H2A) can be bypassed by human TLS DNA polymerases η and κ. However, the authors did not investigate proteolysis of DHCs and the specific proteases involved in their digestion and of the DNA repair pathways that follow TLS. Thus, understanding the role of TLS and associated repair systems in counteracting the genotoxic effects of DHCs requires further investigation.

## The Interplay of DHC Repair Pathways

Due to the heterogeneity and super-bulky size of DHCs, several distinct DNA repair pathways may work in concert to remove them. Although cells lack DHC-specific damage sensors, these lesions can be detected and processed by well-known classic DNA repair pathways with the help of various proteases. When a replication fork is stalled at a DHC, the cross-linked histone could be digested by Spartan/Dvc1 protease and proteasome. Proteolysis of DHC by Spartan and proteasome could be backed up with Ddi1 protease in yeast, or with DDI2 in higher eukaryotes. After proteolysis, the remnant peptide could be bypassed by TLS DNA polymerases to avoid replication fork collapse (Figure 2A). Previous evidence also suggested that partial proteolysis of a DPC/DHC coupled with TLS could be a way to repair these complex DNA lesions (Duxin et al., 2014). However, to avoid TLS induced mutations, proteolysis of DPC/DHC could be coupled to NER and HR. Data from several studies support the idea that the combination of proteolysis and NER could be a convenient error-free mechanism for cells to remove the majority of DPCs. Nonetheless, proteolytic degradation of the protein component may also be coupled with BER during removal of DHC/DPC if NER cannot remove the remnant adduct after proteolysis. Considering that most histone cross-links are formed at abasic sites, the BER pathway may have a specific role in removal of AP site-induced DPCs. This leads us to hypothesize that for the efficient removal of AP site-induced DHCs, proteolysis coupled with BER could be one of the most preferred in cells under genotoxic stress conditions that promote DNA base loss (McKibbin et al., 2013; Prasad et al., 2019; Quiñones et al., 2020). One could speculate that HR proceeds after proteolysis. However, Stingele et al. (2014) have demonstrated that HR and proteolysis are two distinct means of resolving DPCs during the S phase of the cell cycle. When there is a DNA strand incision next to a DPC, or when the DPC load is high, the FA-dependent HR or MRN complex-dependent HR, respectively, may take over the protease-mediated repair. Certain aldehyde-induced DPCs require a DNA strand incision near the lesion, which is further processed by FA-dependent HR. Since reactive aldehydes preferentially induce DHCs, it is proposed that FA-dependent HR (ICL-like repair) may serve as a back-up mechanism when proteolysis coupled TLS/NER/BER is inactive or dysfunctional.

Replication-independent repair of DHCs may involve the 26S proteasome-mediated degradation of DHC, followed by removal of the remnant peptide by the global genome or transcription-coupled (TC) NER (Quievryn and Zhitkovich, 2000; de Graaf et al., 2009) (Figure 2A). Considering that Spartan/Dvc1 protease can play a role outside DNA replication, it may mediate DHC proteolysis that blocks transcription, and the remnants are then removed in the TC-NER sub-pathway. In germ and certain quiescent cells, the GCNA1/ACRC protease may be involved in degradation of DHCs, whereas remnants of the proteolysis could be removed by NER (Figure 2B). In the absence of NER, replication-independent proteolysis by proteasome 26S/Spartan/GCNA1 may be coupled with BER to remove DHCs. These hypothetical models of the interplay of different repair pathways in DHC removal require further studies to test them.

## Concluding Remarks

DNA-protein cross-links occur frequently and are the most bulky DNA lesions in living cells. Among various DPCs, DHCs occupy a special place, because histones, the most abundant DNA-binding proteins, constitute the nucleosome, a basic structural unit of chromatin. Therefore, we propose to classify DNA-histone covalent complexes as a special DHC group of DPCs. Histones cross-link either to DNA bases, mostly guanines and 5-formylcytosines, or to the AP site sugar (Ren et al., 2019). With more than 10,000 abasic sites generated every day in a cell far exceeding the number of oxidized bases (Lindahl, 1993), this lesion is the most abundant type of endogenous DNA damage. Indeed, around 10% of AP sites catalyze the formation of DPCs, among which the majority are likely to be DHCs (Ren et al., 2019). Therefore, it seems that hundreds, and perhaps even thousands of histone cross-links to abasic sites in DNA are formed every day in a cell. Even greater production of DHCs is expected in cells exposed to certain genotoxic stress, leading to significant biological consequences, such as sensitization of cancer cell to chemotherapeutic agents. It is difficult to delineate a single repair pathway for various types of DHCs. Similar to DPCs, the repair of super-bulky DHC lesions requires two steps: partial proteolysis of histone and repair of remaining cross-linked peptide via DNA excision. Cells utilize several proteases and DNA repair pathways for these purposes. Assuming that active BER can generate an excess number of AP sites as repair intermediates of DNA glycosylases, this repair pathway might be one of the major factors in the formation of DHCs in cells. A recent discovery of the role of DNA glycosylases of the Nei-family in repair of bulky ICLs suggests that the BER pathway may participate in removal of AP site-induced DHCs (Couvé-Privat et al., 2007; Semlow et al., 2016; Martin et al., 2017). In eukaryotic cells, chromatin dynamics plays an essential role in DHC removal by various DNA repair pathways. For example, it is well documented that partial and transient nucleosomal DNA unwrapping is indispensable for DNA repair and for transcription initiation. Indeed, several studies have indicated that on average, DNA unwrapping events occur several times per second (Li and Widom, 2004). DHCs should strongly interfere with natural nucleosome unwrapping, and hence with DNA lesion detection, signaling, and repair. Moreover, several laboratories have detected inter-nucleosomal cross-links, including DHCs mediated by histone tail domains. These structures should further impede accessibility of DNA lesions for the DNA repair machinery (Banerjee et al., 2018; Yang and Greenberg, 2019). This implies that PTMs of histone tails via lysine acetylation and methylation, and recruitment of chromatin remodeling complexes, may also influence DHC formation and repair (Yang et al., 2019). Blocking chromatin remodeling and DNA repair pathways involved in DHC removal should lead to the persistence of these DNA lesions and further impede chromatin-based transactions and chromatin organization. However, current knowledge regarding DNA repair pathways involved in DHC removal is far from complete. Further studies are required to delineate the mechanisms involved in the repair of DHCs.

## Author Contributions

MP, BM, MS, and RG wrote the DNA repair part of the review. MP, RG, and AK wrote the proteasome part of the review and prepared figures. All authors discussed and contributed to analysis of published literature and to writing the manuscript. All authors contributed to the article and approved the submitted version.

## Conflict of Interest

AK is the founder and Chief Scientific Officer of InhiProt LLC. The authors declare that the research was conducted in the absence of any commercial or financial relationships that could be construed as a potential conflict of interest.
